# Non-enzymatic glucose sensors composed of trimetallic CuO/Ag/NiO based composite materials

**DOI:** 10.1038/s41598-023-32719-w

**Published:** 2023-04-17

**Authors:** Gowhar A. Naikoo, Mustri Bano, Fareeha Arshad, Israr U. Hassan, Fatima BaOmar, Iman M. Alfagih, Murtaza M. Tambuwala

**Affiliations:** 1 Department of Mathematics & Sciences, College of Arts & Applied Sciences, Dhofar University, 211 Salalah, Oman; 2 College of Engineering, Dhofar University, 211 Salalah, Oman; 3 Department of Pharmaceutics, College of Pharmacy, King Saud University, Riyadh, 4545 Saudi Arabia; 4 Lincoln Medical School – Universities of Nottingham and Lincoln, University of Lincoln, Brayford Pool, Lincoln, LN6 7TS Lincolnshire UK

**Keywords:** Biotechnology, Endocrinology, Medical research, Materials science

## Abstract

The escalating risk of diabetes and its consequential impact on cardiac, vascular, ocular, renal, and neural systems globally have compelled researchers to devise cost-effective, ultrasensitive, and reliable electrochemical glucose sensors for the early diagnosis of diabetes. Herein, we utilized advanced composite materials based on nanoporous CuO, CuO/Ag, and CuO/Ag/NiO for glucose detection. The crystalline structure and surface morphology of the synthesized materials were ascertained via powder X-ray diffraction (P-XRD), energy dispersive X-ray (EDX) spectroscopy, scanning electron microscopy (SEM) and transmission electron microscopy (TEM) analysis. The electro-catalytic properties of the manufactured electrode materials for glucose electro-oxidation in alkaline conditions were probed using cyclic voltammetry (CV) and differential pulse voltammetry (DPV) techniques. Notably, the CuO/Ag/NiO electrode material exhibited exceptional performance as a non-enzymatic glucose sensor, displaying a linear range of 0.001–5.50 mM, an ultrahigh sensitivity of 2895.3 μA mM^−1^ cm^−2^, and a low detection limit of 0.1 μM. These results suggest that nanoporous CuO/Ag/NiO-based composite materials are a promising candidate for early diagnosis of hyperglycemia and treatment of diabetes. Furthermore, non-enzymatic glucose sensors may pave the way for novel glucometer markets.

## Introduction

The scientific communities worldwide have recently given glucose detection approaches a lot of attention^1–4^. Over 537 million people worldwide have diabetes, a chronic disease that claims over 1.5 million lives annually^5^. Most of those affected are from low and middle-income nations, and the prevalence of this disease has only slowly increased over the previous few decades. It is essential to prevent any instances of diabetic emergencies, which happen when blood glucose levels fall below or rise above the typical range between 3.3 and 7.8 mmol L^−1^. Therefore, for diabetic patients to better manage their illnesses and lead healthier lives, it is crucial to develop methods that allow quick and precise blood glucose monitoring^6^.

Scientists worldwide are striving to create highly sensitive, quick, selective, and affordable biosensors to fulfill this need^7–9^. Clark and Updike first created enzyme electrodes and amperometric biosensors in the 1960s^10,11^. Since then, many sensors based on glucose oxidase have been created and have demonstrated excellent specificity and exceptional sensitivity throughout the glucose detection process^12–14^. However, enzyme‒based sensors have shown certain drawbacks, including low repeatability, high instability, being expensive, and requiring complicated manufacturing techniques. Additionally, the efficacy of the enzymatic sensors is impacted by outside variables such as humidity, pH, and temperature variations. The disadvantages consequently constrain the application of enzyme-based sensors^15^. non-enzymatic glucose sensor, on the other hand, have demonstrated remarkable promise of quick detection of glucose molecules to control diabetes^15^.

The interdisciplinary fields of nanoscience and nanotechnology have emerged in the past decade, incorporating material science, bionanosciences, and biotechnology. Metal oxide nanoparticles have great potential in fields such as electronics, magnetics, optoelectronics, information storage, and medication delivery. Nanoparticles have numerous potential uses in areas such as manufacturing, biomedical diagnostics, environmental remediation, and electronics. Metal and metal oxide nanoparticles are considered the most promising agents in various fields due to their high surface volume ratio, with copper and copper oxide nanoparticles demonstrating a range of applications in solving physical, chemical, and environmental problems. The development of nano-biosensors using nanomaterials to improve their properties is highly praised and supported by an increasing number of literature publications, patents, and devices. The combination of nanotechnology and biotechnology has had a significant impact on human life^16–18^.

To address these challenges, researchers have turned to the use of nanoporous materials, which have unique properties such as high surface area, tunable pore size, and enhanced mass transport, making them ideal for use in electrochemical sensing applications. In addition to the challenges associated with enzyme-based and noble metal/alloy-based glucose sensors, the development of a reliable and cost-effective non-enzymatic glucose sensor has also posed its own set of challenges. One of the main obstacles in non-enzymatic glucose sensing is achieving high selectivity towards glucose molecules in the presence of other interfering species commonly found in biological fluids. Moreover, non-enzymatic glucose sensors have also suffered from issues with sensitivity, stability, and reproducibility^12^. The literature review provides an overview of the research work carried out in the field of nonenzymatic glucose sensing using advanced nanomaterials. The studies discussed in the review demonstrate that the use of nanomaterials, such as reduced graphene oxide (rGO), Cu_2_O nanoclusters, Pd-CuO nanoparticles (NPs), and Au-CuO NPs decorated rGO, can significantly improve the sensitivity, detection limits, and linear ranges of glucose sensors. The authors also highlight the challenges that need to be addressed to improve the performance and stability of nanomaterial-based glucose sensors. Overall, the review emphasizes the potential of nanomaterials for developing efficient and accurate glucose monitoring systems for diabetes management^19–23^.

**Scheme 1:**
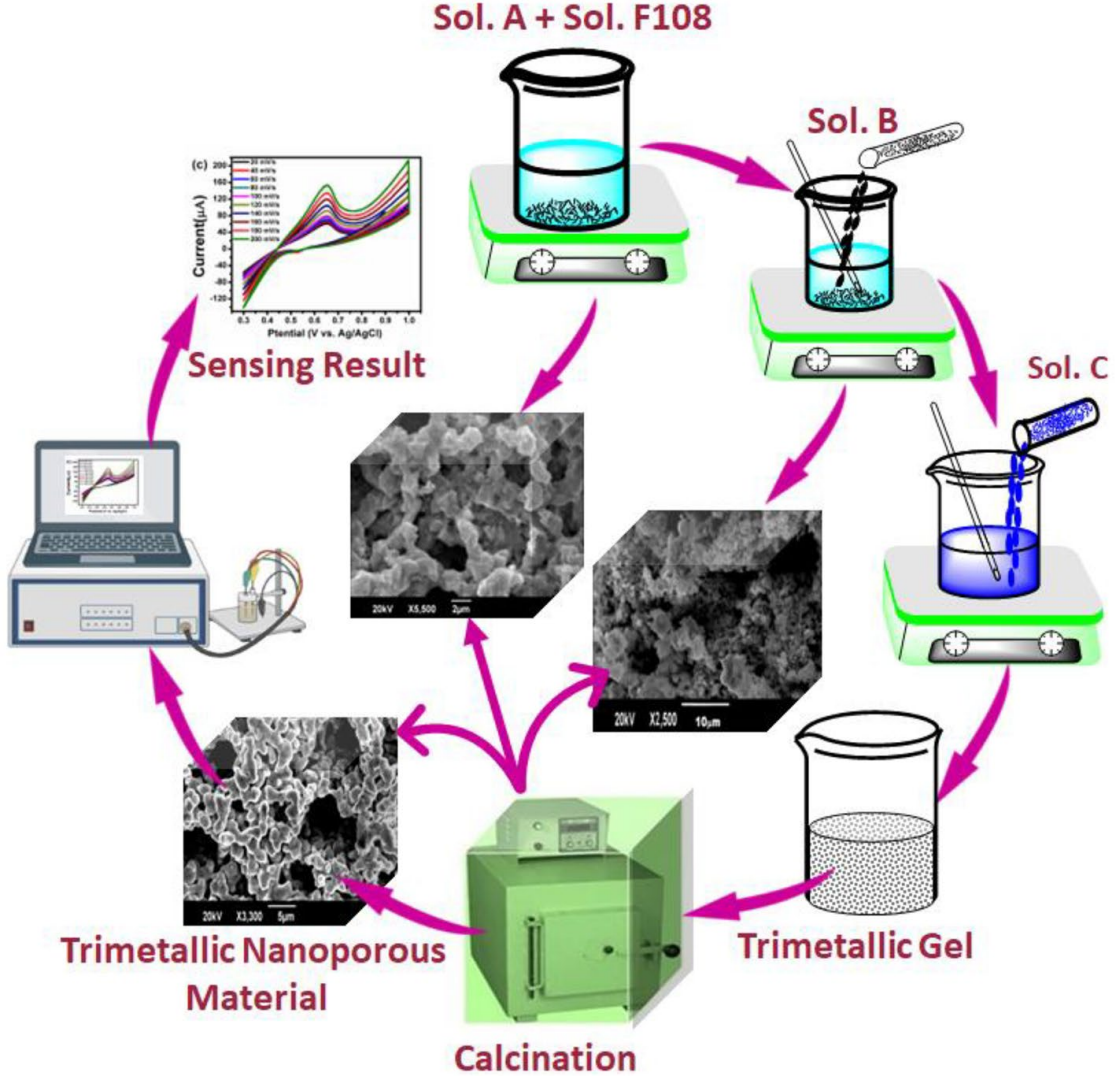
Schematic representation of glucose sensing.

The use of nanoporous materials in glucose sensing has shown great promise, with several studies reporting high sensitivity, selectivity, and stability of nanoporous materials towards glucose detection. The current study focuses on the synthesis and electrochemical characterization of nanoporous CuO, CuO/Ag, and CuO/Ag/NiO-based composite materials for non-enzymatic glucose sensing. The use of transition metals and their oxides as a viable alternative to noble metal-based materials offers a more sustainable and cost-effective solution for the development of glucose sensors. The use of bimetallic and trimetallic compositions in the fabrication of nanoporous materials provides the opportunity to enhance the selectivity and sensitivity of the sensors towards glucose molecules. The electrochemical characterization of the synthesized nanoporous materials using cyclic voltammetry and differential pulse voltammetry techniques has shown excellent performance in terms of selectivity, stability, and accuracy, with a low detection limit of 0.1 M and a linear detection upper limit of 0.001 mM. These results demonstrate the potential of the synthesized nanoporous materials as promising candidates for the development of commercial glucose sensors and other electrochemical sensing applications (Scheme 1).

## Methods

### Synthesis of nanoporous CuO, bimetallic CuO/Ag and trimetallic CuO/Ag/NiO composite materials

In a 100 mL beaker, 2 g of Cu(NO_3_)_2_ (57.14 wt% of, BDH) was dissolved in 1.5 g of ultrapure water (42.85 wt%) and added to 2 g of Synperonic® F 108 (average Mn 14,600, Sigma–Aldrich, 17.39 wt%) in 11.5 g of water (82.60 wt%) and stirred for 30 min to generate a gel that finally turned into a dark blue colored paste. The hydrophilic part of Synperonic® F 108 (non-ionic surfactant) reduces Cu(NO_3_)_2_ to Cu and oxidize Cu to CuO. The resulting gel was stored at normal temperature for 120 h before being calcined for 5 h at 500 °C with a heating/cooling rate of 4.17 °C/min to room temperature in a furnace.

For the preparation of bimetallic CuO/Ag, 2 g of Cu(NO_3_)_2_(57.14 wt%) was mixed with 1.5 g of water (42.85 wt%), 4 g of Synperonic® F 108 (4.03 × 102 M, 14.82 wt%, suspension in 85.18 wt% of ultrapure water, Sigma–Aldrich), and 2 g of AgNO_3_(57.14 wt%, Sigma–Aldrich) dissolved in 1.5 g of ultrapure water were added, followed by the ageing and calcination as aforementioned.

Trimetallic CuO/Ag/NiO gel was synthesised by combining 2 g of Cu(NO_3_)_2_(57.14 wt%, suspension in 42.86% water) with 6 g of Synperonic® F 108 (4.03 × 102 M, 15.00 wt %, suspension in 85.00 wt% water, Sigma–Aldrich) as well as 2 g of AgNO_3_ (57.14 wt%, suspension in 42.86% water), and 2 g of Ni(NO_3_)_2_ (57.14 wt%) in 1.5 g of ultra–pure water (42.85 wt%). The above materials were then aged and calcined as aforementioned^24–26^.

### Materials characterization

X Pert PRO X–Ray Diffraction with Cu Ka radiation was used for the P–XRD analysis. A JEOL JSM–6510LA electron microscope was used to explore SEM and EDX studies. A TEM study was explored by using a JEOL Model: no. JEM1400 instrument. MUFFLE FURNACE, MODEL: HD150 PAD was used for the synthesis of all the materials. CV and DPV studies were carried out by Autolab PGSTAT204 FRA32M.

### Preparation of modified non-enzymatic glucose sensor electrodes

All electrochemical experiments were conducted at room temperature with three electrodes: an Ag/AgCl reference electrode, a Pt counter electrode, and a glassy carbon working electrode (GCE, diameter 3.0 mm). Working electrodes were synthesized by modifying the bare–glassy carbon electrode (b–GCE) surface with a dispersal of CuO–GCE, CuO/Ag–GCE and CuO/Ag/NiO–GCE. The corresponding dispersion from the aforesaid synthesised monoliths was included by ultrasonically dissolving 5 mg of each monolith individually in 10 mL of ethanol. Before the electrode modification, the GCE surface was rigorously polished with alumina slurry, cleaned with sonication in ethanol for 10 min, washed with distilled water, dried at room temperature, and lastly cleaned again. The dispersed mixture was poured in 10 μL over through the surface of the GCE, and thus the solvent was permitted to evaporate at room temperature. For electrode optimization, the electrochemical activation of modified GCEs was performed using cyclic potential sweeps in the range of − 1.0 to + 2.0 V in 0.1 M nitric acid solution to obtain a steady voltammogram. Following each electrochemical study, the electrode surface was washed by conducting cyclic sweeps in the opposite direction (1.0 V to 0.0 V) in 1 mM NaOH.

## Results

### Powder X-ray diffraction (P-XRD) and energy dispersive X-Ray (EDX) spectra

**Figure 1:**
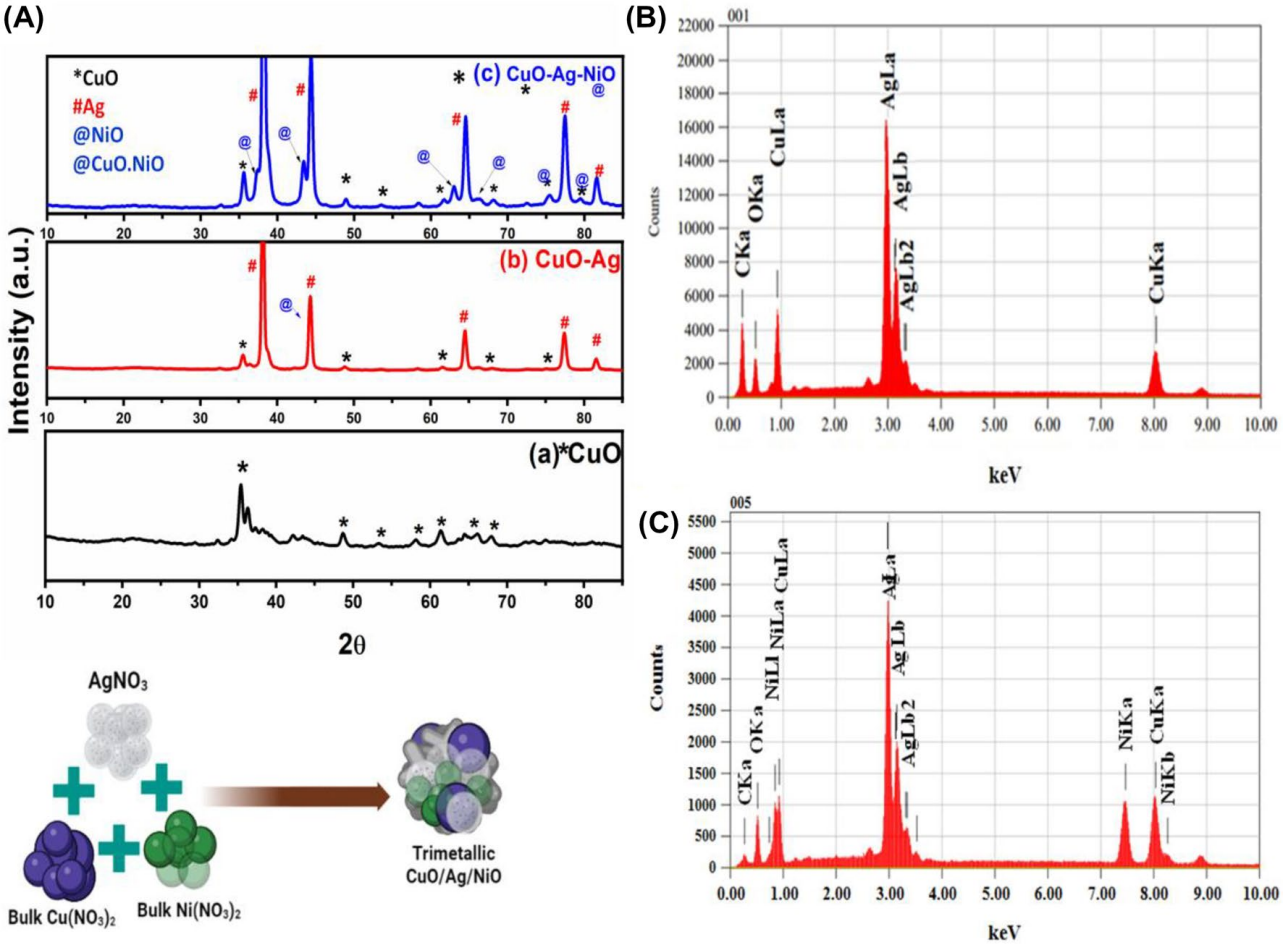
P–XRD result of (**A**) (a) CuO, (b) CuO/Ag, (c) CuO/Ag/NiO; EDX results of (**B**) CuO/Ag, and (**C**) CuO/Ag/NiO.

The crystalline structure and crystallinity of the synthesized materials were analyzed using P-XRD, and the results were presented in Fig. 1Aa–c. The crystalline structure and crystallinity of the materials synthesised were investigated using P–XRD. The XRD patterns are shown in Fig. 1Aa–c for CuO, CuO/Ag, and CuO/Ag/NiO, respectively. The diffraction patterns showed that the synthesized materials exhibited cubic phase of CuO corresponding to (111), (200), (202), (113), (311) and (220) planes at 2θ values of 35.37°, 43.22°, 48.50°, 62.72°, 65.87° and 68.19° (JCPDS No. 48–1548). In Fig. 1Ab new peaks appear in the P-XRD data of CuO/Ag at 38.21°, 44.41°, 64.46°, 77.40° and 81.49° corresponding to (111), (200), (220) and (311) planes exhibited FCC phase can be because of the presence of silver. In Fig. 1Ac, the result shows the presence of nickel oxide peaks appear at 37.14°, 43.33°, 63.28°, 75.61° and 79.55° along with CuO and Silver and (111), (200), (220), (311), and (222) crystal planes exhibit FCC phase. The absence of additional peaks confirms the purity of CuO, CuO/Ag and CuO/Ag/NiO nanoporous materials (Fig. 1Aa). Thus, the XRD results confirmed the optimized experimental conditions for the preparation of nanoporous CuO, CuO/Ag and CuO/Ag/NiO.

The elemental composition and distribution of CuO, CuO/Ag, and CuO/Ag/NiO nanoporous materials were analyzed using EDX. The results of the EDX analysis for CuO/Ag are shown in Fig. 1B, revealing the presence of copper (Cu), silver (Ag), and oxygen (O) in the synthesized sample. The analysis showed that the as-synthesized material was almost stoichiometric, with a weight percentage of Cu (71.44%), O (5.20%), and Ag (23.16%) as a minor phase with a trace impurity of carbon (C). The absence of other elemental impurities was also confirmed in the EDX spectra. The EDX spectra for CuO/Ag/NiO depicted in Fig. 1C showed significant peaks for copper, silver, Nickel and oxygen, which is consistent with the XRD graph in Fig. 1Ac. The weight percentage for oxygen, copper, silver, and nickel was 1.60, 21.35, 53.66, and 16.42%, respectively. Additional peaks observed in the EDX spectra were due to impurities from carbon (C) in the substrate. Therefore, the EDX analysis confirmed the successful synthesis of CuO/Ag and CuO/Ag/NiO nanoporous materials.

### Scanning electron microscope (SEM) and transmission electron microscopy (TEM) analysis

**Figure 2:**
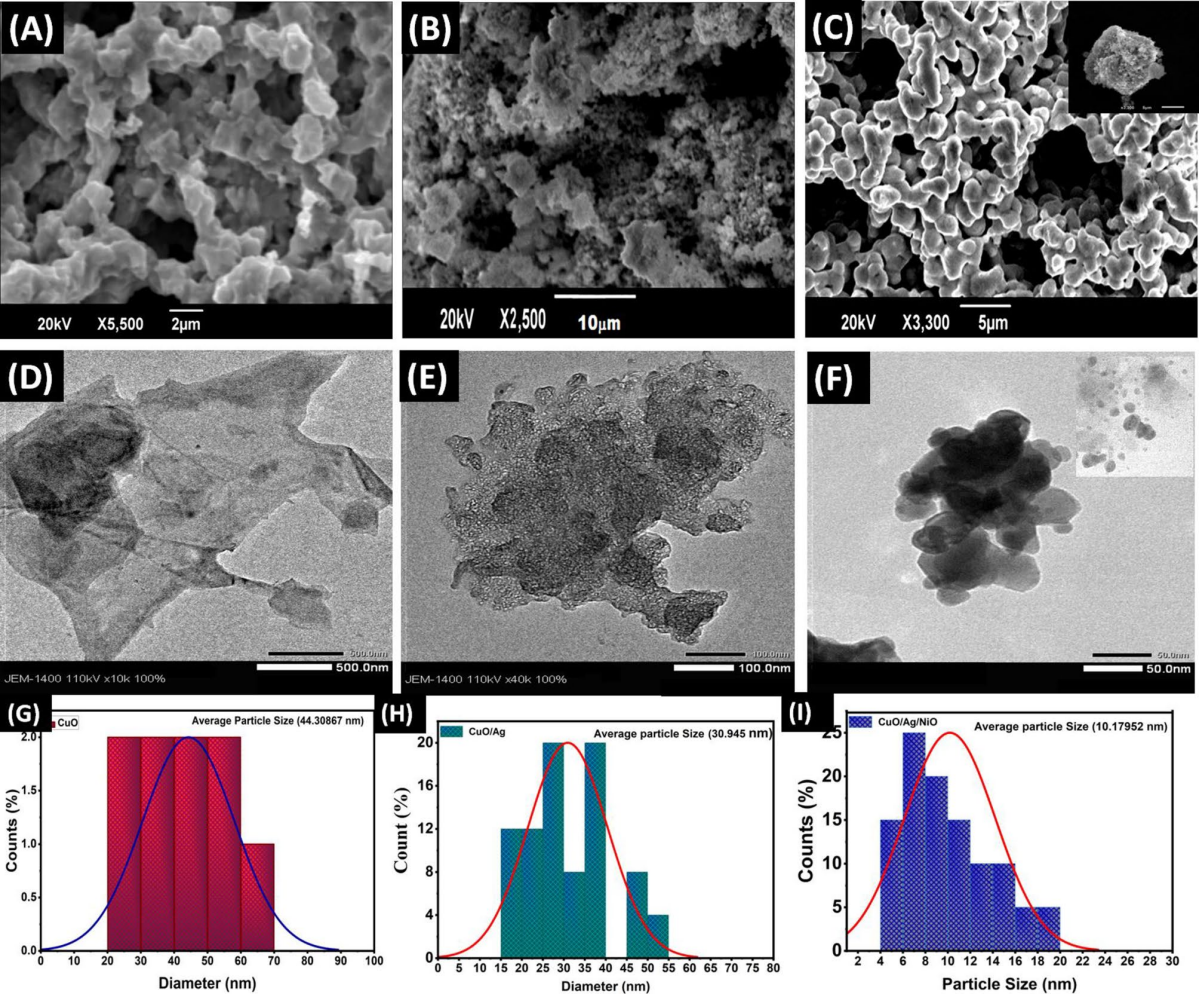
SEM images of (**A**) CuO, (**B**) CuO/Ag, (**C**) CuO/Ag/NiO, and inset image shows the agglomeration of CuO/Ag/NiO; TEM images of (**D**) CuO, (**E**) CuO/Ag, and (**F**) CuO/Ag/NiO, The diameter distribution of (**G**) CuO, (**H**) CuO/Ag and (**I**) CuO/Ag/NiO.

The surface morphology of the synthesised samples and the changes in the composite materials that occur upon adding Ag and NiO were studied using SEM. The SEM images for CuO, CuO/Ag, and CuO/Ag/NiO are shown in Fig. 2A–C respectively. The SEM image in Fig. 2A shows that the particles are porous, well-spaced, and possess polyhedron shape a well-defined uniform crystalline structure of the CuO particles. When Ag was added to CuO, the morphology changed to a distinct nanovesicle-shaped porous structure, as depicted in Fig. 2B. The image demonstrates that the size of the pores in the CuO has decreased, which could be related to the deposition of silver particles in the pores of the CuO. Silver particles are known to be deposited in the pores of CuO due to their relatively large size compared to that of the pores, leading to a decrease in the pore width. This decrease in pore size is likely due to the silver particles blocking the pore openings, reducing the size of the pore and preventing further particle passage. Furthermore, upon adding NiO to CuO/Ag, the particles of the compound achieved a more agglomerated form, with increasing the space between the particles, as shown in Fig. 2C. This is because of the electrostatic interactions between the particles, which make them more firmly attracted to one another and create a clearer, more prominent porous structure in the shape of a nanovesicle. This can result in a more stable form of the compound, which can be beneficial for certain applications. This could be because of the deposition of the NiO within the previously smaller pores observed in the CuO/Ag structure (Fig. 2C inset).

The TEM images confirming the size and shape of the synthesised CuO, CuO/Ag, and CuO/Ag/NiO particles are shown in Fig. 2D–F. As shown in Fig. 2D, the CuO nanostructures appear porous, highly agglomerated, and possess predominantly spherical shapes in the range of 500 nm. Figure 2E shows the TEM image of CuO/Ag nanoparticles in the range of 100 nm. Certain areas on the image appear darker than the rest, showing the presence of Ag particles with uniform thickness and considerable dispersion over the CuO surface. This means that because of the presence of Ag particles, the distribution and the stability of the CuO surface have increased to prevent further agglomeration of the material. Furthermore, Fig. 2F shows the TEM image of the synthesized CuO/Ag/NiO particle at the scale of 50 nm. The particles appear as dark spots in the image, with the darkness of each spot corresponding to the amount of each material present in the particle. A large cluster of CuO, Ag, and NiO of dimensions below ~ 50 nm in Fig. 2F was observed. The particles appear to overlap each other and display a roughly three-dimensional, spherical shape. The diameter distribution graphs for CuO, CuO/Ag, and CuO/Ag/NiO nanoporous images are shown in Fig. 2G–I respectively.

### Electrochemical performances

**Figure 3:**
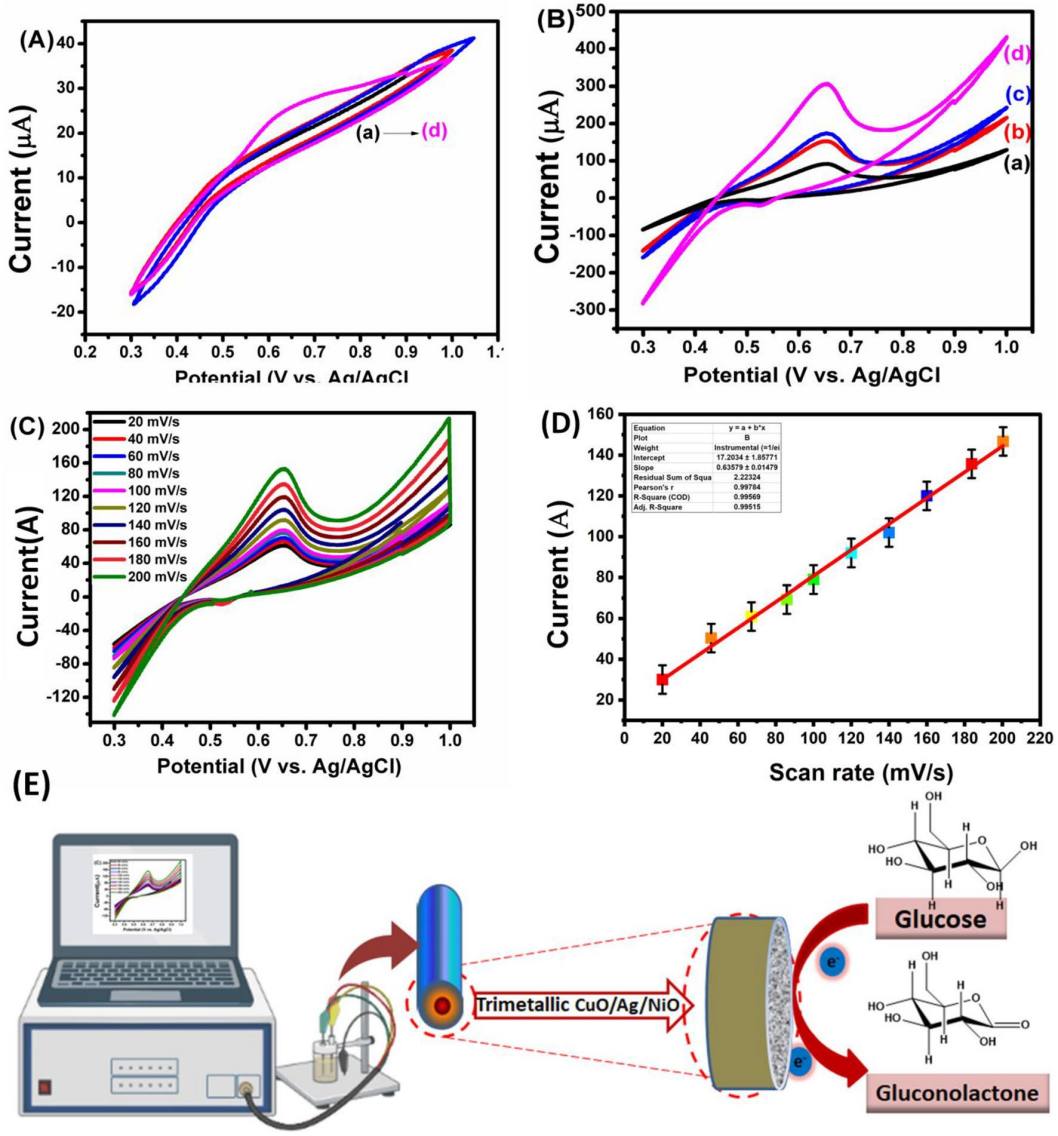
(**A**) Typical CV response of electrodes (a) b–GCE, (b) CuO–GCE, (c) CuO/Ag–GCE, and (d) CuO/Ag/NiO–GCE in NaOH solution at scan rat, 100 mVs^−1^, (**B**) CV curve of different electrodes (a) b–GCE, (b) CuO–GCE, (c) CuO/Ag–GCE, (d) CuO/Ag/NiO–GCE in presence of glucose at the various electrodes (scan rate, 100 mVs^−1^), (**C**) The CV curve of CuO/Ag/NiO–GCE in 0.1 mM glucose at scan rates (20–200 mVs^−1^), and (**D**) calibrated peak current vs. scan rate graph. (**E**) Schematic representation of trimetallic electrode for producing glucose to gluconolactone.

The electrochemical performances of the developed nanoporous electrodes were investigated by CV performance in 0.1 M NaOH at a scan rate of 100 mVs^−1^, as shown in Fig. 3A. The CV obtained using the b–GCE, CuO–GCE, and CuO/Ag–GCE in the blank NaOH solution showed no visible redox behavior proving that the electrodes are potentially inactive under alkaline conditions. CuO/Ag/NiO–GCE in presence of blank NaOH solution showed small oxidation peak observed at 0.6 V is attributed to the oxidation of NiO behavior proving that the electrodes are potentially active under alkaline conditions. Thus, the synthesised porous CuO/Ag/NiO structures exhibited great electro‒catalytic activity under alkaline conditions and exhibited the chemical reaction as mentioned below:

$$CuO + H_{2} O + 2OH^{ - } \to Cu\,\left( {OH} \right)_{4} + 2e^{ - }$$

$$Cu\left( {OH} \right)_{2} + OH^{ - } \to CuO\,\left( {OH} \right) + H_{2} O + e^{ - }$$

$$NiO + H_{2} O + 2OH^{ - } \to Ni\,\left( {OH} \right)_{4} + 2e^{ - }$$

$$Ni\left( {OH} \right)_{2} + OH^{ - } \to NiO\,\left( {OH} \right) + H_{2} O + e^{ - }$$

### Electro-catalytic oxidation of glucose

The electro-oxidation of glucose at b–GCE, CuO‒GCE, CuO/Ag–GCE, and CuO/Ag/NiO–GCE was studied by CV. 2 mM glucose was added to 0.1 M NaOH and was studied using CV at a scan rate of 100 mVs^−1^. As shown in Fig. 3B, the voltammograms illustrate that the bare electrode showed no significant peak. However, a modest current was detected for glucose oxidation, with negligible interaction towards the electro oxidation of glucose in 0.1 M NaOH across the specified potential window. On the other hand, the surface area of the CuO-GCE, CuO/Ag-GCE, and CuO/Ag/NiO-GCE modified electrodes was calculated to be 1.72, 1.58, 0.32 cm^2^. CuO/Ag/NiO-GCE modified electrode displayed an Ipa of 300 μA at + 0.65 V versus Ag/AgCl. This is because of the glucose oxidation involving the Cu^2+^ and Ni^2+^ centers in 0.1 M NaOH solution. The electro-chemical reaction possibly happens as per the chemical equation mentioned below:

$$glucose +CuO\left(OH\right)\to gluconolactone+{Cu\left(OH\right)}_{2} +{H}_{2}O$$

$$glucose +NiO\left(OH\right)\to gluconolactone+{Ni\left(OH\right)}_{2} +{H}_{2}O$$

The oxidative peak current was significantly enhanced when CuO/Ag/NiO–GCE was further analyzed, as shown in Fig. 3C, demonstrating its larger Ipa value of 157 A at + 0.65 V compared to Ag/AgCl at a scan rate of 200 mVs^−1^. This improvement is related to the intense interaction between CuO, Ag, and NiO particles. The effective surface area is further increased, incorporating the Ag and NiO particles provide a more active glucose oxidation site. The porous CuO/Ag/NiO–based electrode surface also promotes electron transport, resulting in quick glucose diffusion and adsorption. Here, the oxidation of glucose molecules to glucolactone causes a massive increase in current. Compared to bare, CuO, CuO/Ag and CuO/Ag/NiO-based electrodes showed better electrochemical activity towards glucose oxidation. However, CuO/Ag/NiO‒based electrode displayed higher electrochemical activity towards glucose oxidation than the bare, CuO or CuO/Ag-based electrodes. As demonstrated in Fig. 3D, the effect of scan rate on the response curve of glucose oxidation using 2 mM glucose in 0.1 M NaOH was also investigated. It was observed that as the scan rate increased, the oxidative current increased too. With an excellent correlation value of 0.9956, oxidative current (Ipa) demonstrated direct proportionality with the scan rate, reflecting that glucose oxidation is an adsorption-controlled electrode process. Schematic representation also presents a clear view of trimetallic electrode for producing glucose to gluconolactone in Fig. 3E.

### Amperometric study of glucose

**Figure 4:**
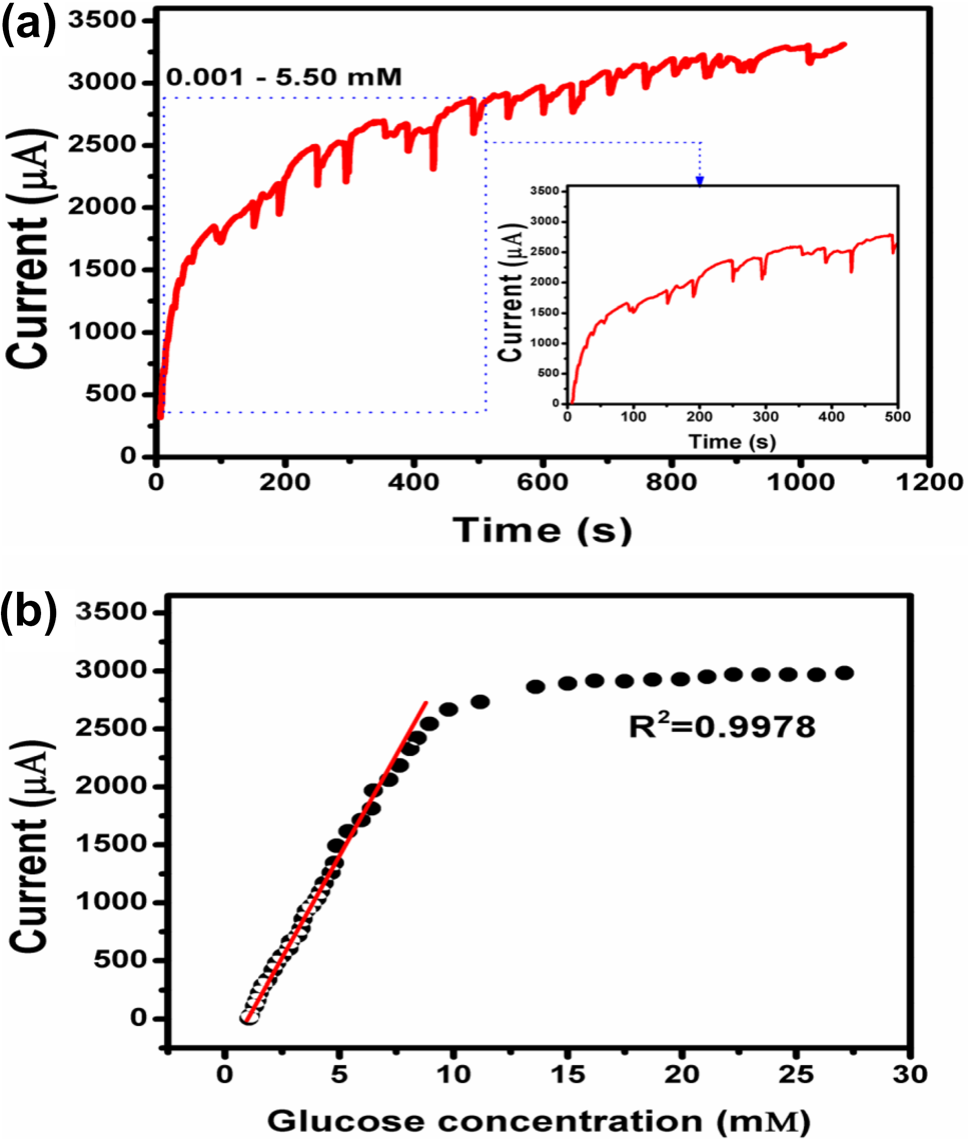
(**a**) Amperometric result of CuO/Ag/NiO–GCE at + 0.58 V in 0.1 M NaOH solution with various glucose concentrations from 0.001 to 5.50 mM and inset response curve shows the low concentration range of glucose (0.001–2.95 mM). (**b**) Plot illustrating the current response vs. glucose concentration.

With successive stirring and glucose concentrations varying from 0.001 to 5.50 mM in 0.1 M NaOH, Fig. 4 shows the amperometric response across the CuO/Ag/NiO–GCE electrode at + 0.58 V versus Ag/AgCl. The inset response curve in Fig. 4 provides a clearer view of the low glucose concentration between 0.001 and 2.95 mM. At higher potential, more electro-active molecules quickly oxidize and interact with the electrode and thus affect the precision of glucose measurements. Moreover, the absorption of intermediates is irreversible, damages the electrodes and prevents glucose oxidation. Hence, + 0.58 V was chosen as the ideal operating voltage. The amperometric response of the CuO/Ag/NiO–GCE electrode was tested upon the rapid addition of glucose, as shown in Fig. 4a. Even at low glucose concentrations between 0.001 to 5.50 mM, a quick response time of only 0.8 s was seen (see the inset of Fig. 4a). The matching calibration curve, which depicts a linear adjustment between the current density and glucose concentration, is shown in Fig. 4b. The CuO/Ag/NiO–GCE electrode appears to be very effective in detecting changes in glucose levels over a wide concentration range based on stepwise linear increments. By dividing the slope of the linear component of the calibration curve by the electrode surface area, the sensitivity was determined to be 2895.3 μA mM^−1^ cm^−2^. Also, a low detection limit of 0.1 μM was observed at the signal-to-noise ratio (S/N) of 3.

### Mechanism of action

Furthermore, the ultrahigh sensitivity CuO/Ag/NiO–GCE to glucose detection is revealed by the linear increase in anodic peak current density with increasing glucose loading. The increased current density is primarily due to the increased number of active sites and a larger surface area exposed to glucose. The large surface area and high number of active sites enable a rapid electron transfer, which is responsible for the high sensitivity to glucose detection. The CuO/Ag/NiO–GCE electrode also has a low over-potential, which contributes to its high sensitivity. This means that the material requires less energy to generate an electron transfer, resulting in a lower detection limit. Additionally, the CuO/Ag/NiO–GCE electrode has a low charge–transfer resistance, which further increases its sensitivity. This indicates that the material has a low resistance to electron flow, resulting in rapid electron transfer, leading to increased current density.

**Figure 5:**
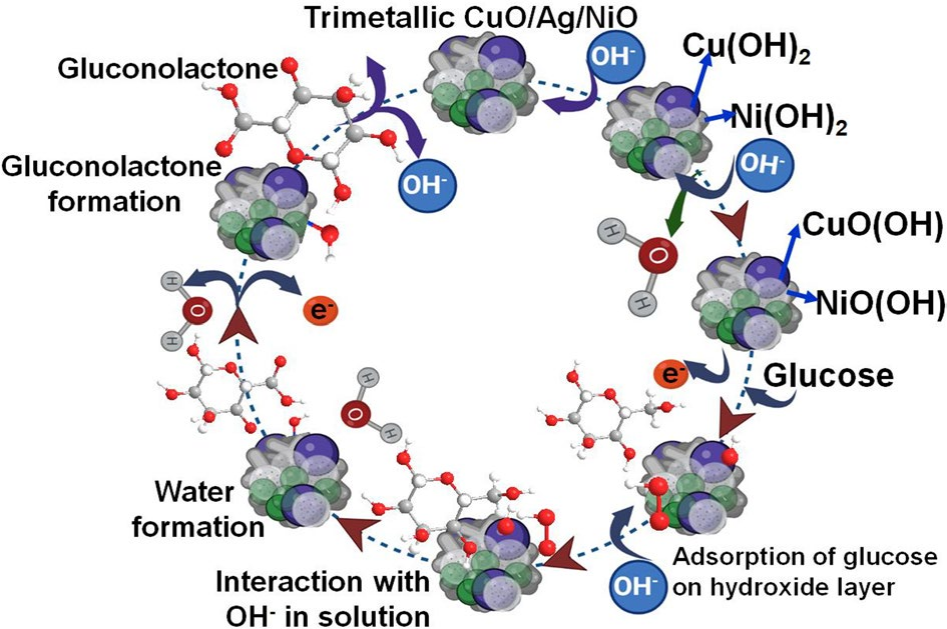
Mechanism of the trimetallic electrode for producing glucose to gluconolactone.

When the modified electrode is immersed in the NaOH solution, the CuO layer acts as the catalytic layer and facilitates the oxidation of glucose. The Ag layer acts as a charge transfer layer, which helps in the electron transfer between the CuO and NiO layers. The NiO layer acts as the active layer and serves as the site where the glucose molecules are oxidized. The mechanism of action of the trimetallic electrode for producing glucose to gluconolactone is described below in Fig. 5. The process starts with the CuO/Ag/NiO–GCE surface forming a monolayer of Cu(OH)_2_ and Ni(OH)_2_ on the surface during the first sweep. Cu(OH)_2_ and Ni(OH)_2_ then undergoes further oxidation to CuO(OH) and NiO(OH) in an alkaline medium. The glucose molecules in solution then adsorb onto the CuO/Ag/NiO surface, forming a monolayer of glucose molecules on the surface.

The next step involves the monolayer of glucose molecules undergoing an oxidation reaction, catalyzed by the CuO/Ag/NiO–GCE, resulting in the formation of gluconolactone. Finally, the oxidized CuO(OH) and NiO(OH) is electrochemically oxidized back to Cu(OH)_2_ and NiO(OH)_2_, completing the process. This mechanism demonstrates that the CuO/Ag/NiO–GCE material has low electron flow resistance, resulting in faster electron transfer with higher current density. Throughout the process, CuO and NiO acts as an electron transfer in an alkaline medium, starting with its oxidation to CuOOH and NiOOH. As a result, glucose is oxidized to gluconolactone, and CuOOH and NiOOH is concurrently reduced to CuO and NiO after accepting the electrons from glucose (Fig. 5). This mechanism highlights the importance of the material used in the electrochemical glucose oxidation process and how it can affect the efficiency and performance of the electrodes. Understanding this mechanism can help in the development and optimization of more efficient and reliable electrochemical glucose sensing devices.

The experimental results indicate that the combined reactivity of CuO/Ag/NiO–GCE, which are characterized by different atomic structures, influences the overall performance of the nanoflower in the sensing application.

### Anti-interference ability test

**Figure 6:**
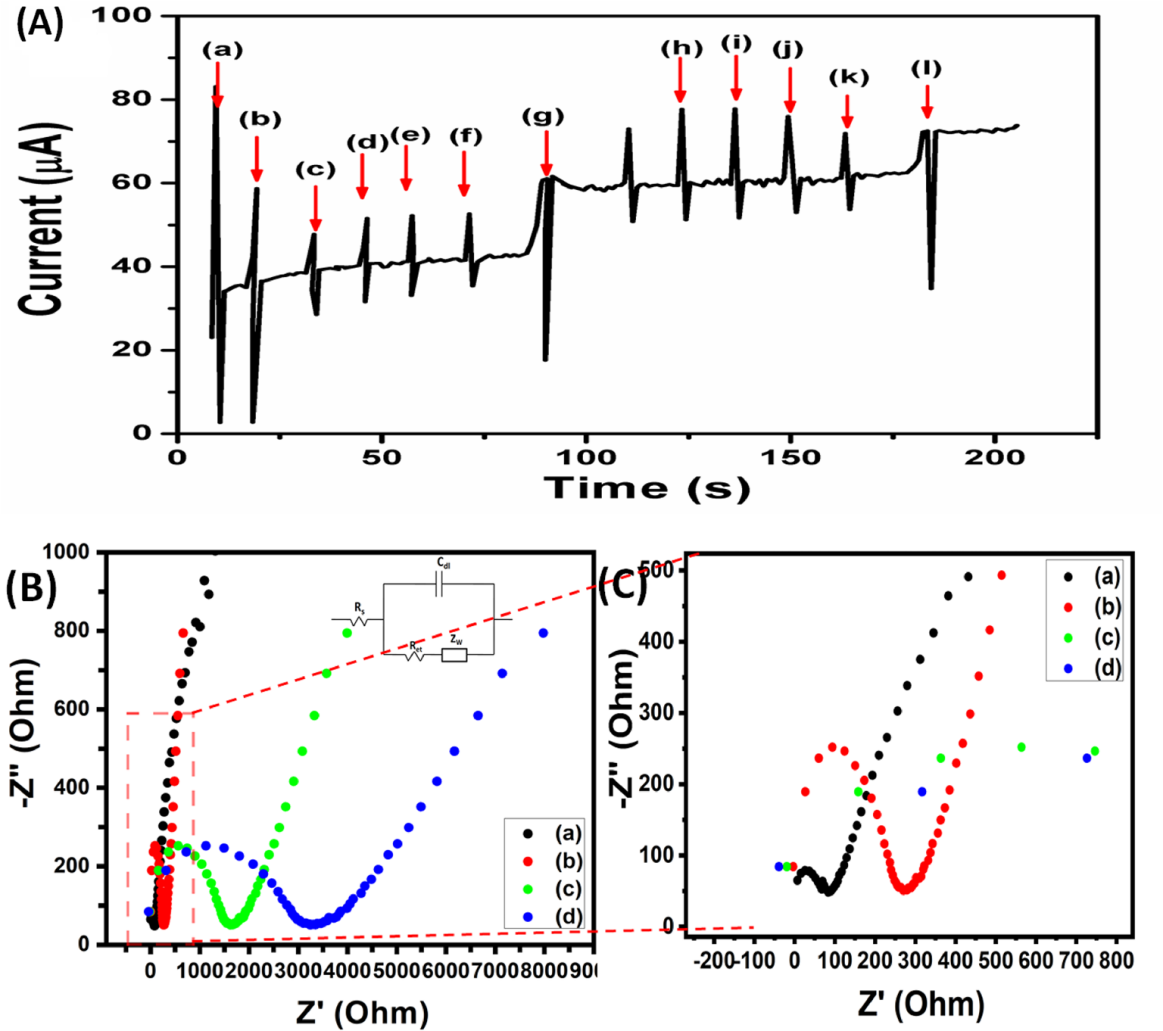
(**A**) Illustrates the amperometric result of the CuO/Ag/NiO -GCE electrode to the sequential addition of 0.1 mM glucose and 0.1 M NaOH solution of 0.01 Mm potential interfering species, including (a) glucose (b) AA, (c) DA, (d) UA, (e) Cys, (f) Ni^2+^, (g) Ca^2+^, (h) Mg^2+^, (i) lactose, (j) sucrose, (k) maltose, and (l) mannose at + 0.58 V (versus Ag/AgCl). (**B**) A representative Nyquist plot shows semicircle EIS spectrum in a solution of 5 mM [Fe(CN)_6_]^3−/4−^ and 0.1 M KCl mixtures at an applied amplitude of ± 5 mV over a frequency range (0.01 Hz to100 MHz) of (a) CuO/Ag/NiO–GCE (b) CuO/Ag–GCE (c) CuO–GCE (d) b–GCE. (**C**) Clear zoom view of Nyquist plot shows clear semicircles EIS spectrum graph of (a) CuO/Ag/NiO–GCE and (b) CuO/Ag–GCE.

Several oxidation species are simultaneously present in human blood. Fourth generation glucose sensing devices have significant difficulty in detecting interference, which could potentially affect the electrode's accuracy in perceiving glucose levels. For a sensor to be highly accurate while giving results, it has to be highly selective. The selectivity of the sensors indicates that their applicability in detecting analytes in the actual sample. Biomolecules like D-fructose possess closely similar electrical activity to glucose molecules. Also, other molecules like uric acid (UA), dopamine (DA), and ascorbic acid (AA) are present along with glucose in serum samples^27^. Therefore, we kept the ratio of potential interference to glucose close to 1:10, higher than the approximate ratio under physiological conditions. Figure 6A illustrates the amperometric result of the CuO/Ag/NiO–GCE electrode to the sequential addition of 0.1 mM glucose and 0.1 M NaOH solution of potential interfering species, including (a) glucose (b) AA, (c) DA, (d) UA, (e) Cys, (f) Ni^2+^, (g) Ca^2+^, (h) Mg^2+^, (i) lactose, (j) sucrose, (k) maltose, and (l) mannose. The results indicate that current responses produced in the presence of interfering species are nonexistent compared to those produced in the existence of glucose molecules. Moreover, even in the existence of interfering molecules, the current signals produced for glucose remained unique and unambiguous. The developed electrodes were further studied using electrochemical impedance spectroscopy (EIS) in a solution of 5 mM [Fe(CN)_6_]^3−/4−^ and 0.1 M KCl mixtures at an applied amplitude of ± 5 mV over a frequency range (0.01 Hz to100 MHz) for glucose detection and evaluation. The Nyquist diagrams of the CuO/Ag/NiO–GCE, CuO/Ag–GCE, CuO–GCE, and b–GCE are shown in Fig. 6B. Figure 6C shows a clear zoom view of Nyquist plot presenting clear semicircles EIS spectrum graph of (a) CuO/Ag/NiO–GCE and (b) CuO/Ag–GCE for better understanding. Wide linear range of 0.001–5.50 mM, a correlation value of 0.9978, and a LOD of 0.1 μM were also features of the developed non-enzymatic glucose sensor. Additionally, Table 1^28–41^ compares the sensing performance of present modified sensing electrode to other non-enzymatic glucose sensors that have been previously published. CuO/Ag/NiO–GCE demonstrated great sensitivity (2895.3 μA mM^−1^ cm^−2^) in a broad linear range (0.001 to 5.50 mM), which is higher than previously reported values for glucose sensing, as shown in the table.

**Table 1:** Analytical performance comparison of non-enzymatic glucose sensor for diabetes monitoring.

Electrode	Sensitivity	Linear Range	Response time	LOD	References
CuO/Ag/NiO–GCE	2895.3 μA mM^−1^ cm^−2^	0.001–5.50 mM	0.8 s	0.1 μM	This work
Ag-doped CuO microflowers on MLG	1527 μA mM^−1^ cm^−2^	0.01 mM ~ 6.0 mM	–	–	^28^
Cu_2_O@CuO nanosheets	818.5 μA mM^−1^ cm^−2^	0.02 to 5 mM	–	8.75 μM	^29^
Ni–Cu LDH@Cu(OH)_2_ NWs/CuF	7.08 μA μM^−1^ cm^−2^	0.006–1.6 μM	–	1.3 μM	^30^
CNTs/CuO	15.3 mA cm^−2^ µM^−1^	5–100 µM	2 s	3.90 µM	^31^
SWCNTs/Cu_2_O/ZnO–NRs/graphene	466.1 μA mM^−1^ cm^−2^	11.1 mM	< 1 s	–	^32^
CuNPs–LIG	495 μA mM^−1^ cm^−2^	0.001–6 mM	< 0.5 s	0.39 μM	^33^
Cu/rGO/SPE	172 μA mM^−1^ cm^−2^	0.10–12.5 mM	35 s	65 μM	^34^
Cu–MS	0.25 mA cm^–2^ mM^–1^	1 μM and 3 mM	–	1.75 μM	^35^
CuO–U	0.32 mA cm^–2^ mM^–1^	1 μM and 3 mM	–	7.56 μM	^35^
CuO	904 μA mmol^−1^ L^−1^ cm^−2^	0.5–5.0 mmol L^−1^	–	0.002 mmol L^−1^	^36^
Cu_2_Se	15.341 mA mM^−1^ cm^−2^	0.25 μM − 8 mM	–	0.26 μM	^37^
Cu_2_O ordered nanoarrays	–	0.05 to 15 mM	–	–	^38^
Cu/Cu_2_O NPs	28,071 uA mM^−1^ cm^−2^	Up to 7.8 mM	< 1 s	2.46 μM	^39^
NP–Cu_2_O/CuO NWA/GCE	1950 μA mM^−1^ cm^−2^	0.1–6 mM	1.5 s	1 μM	^40^
CuSe	19.419 mA mM^−1^ cm^−2^	100 nM–40 µM	< 2 s	0.196 µM	^41^

### Reproducibility, stability, and reusability tests

Five modified sensor electrodes made under identical conditions were used to examine the amperometric current responses of glucose at 0.3 V, which allowed the CuO/Ag/NiO–GCE sensors repeatability to be studied. Additionally, 20 further successive amperograms were taken using the same electrode. The relative standard deviation was less than 2%, demonstrating the outstanding reproducibility and repeatability of the CuO/Ag/NiO–GCE sensor. By using the developed electrode for 4 weeks, the stability of the electrode was evaluated. The electrode showed 96% of their initial current responses, indicating that the CuO/Ag/NiO–GCE sensor has an acceptable level of stability. During these trials, the monolith was actively attached to the GCE surface. Before repetition, it was always washed and electrochemically activated.

## Conclusion

In conclusion, we have successfully synthesized and characterized nanoporous CuO, CuO/Ag, and CuO/Ag/NiO-based composite materials and investigated their electrochemical performance as potential candidates for glucose sensors and other applications that require quick electrochemical activity. Our results show that the synthesized CuO/Ag/NiO–GCE showed a rapid electron transfer, proving a significant ability towards the quantification of glucose sensing up to the lowest limit (0.1 μM), an ultrahigh sensitivity of 2895.3 μA mM^−1^ cm^−2^ and a linear range of 0.001–5.50 mM. The remarkable electrochemical performance of these materials is attributed to their unique structural and morphological properties, including high surface area, well-defined pore structures, and the presence of multiple metal components. The synergistic effect of these properties results in enhanced electrocatalytic activity, making these materials promising candidates for use in a wide range of electrochemical applications. Furthermore, the synthesis of these nanoporous composite materials opens up new avenues for developing advanced materials with improved performance for various applications. The successful synthesis of these materials and the results obtained in this study provide a strong foundation for further research and development in the field of electrochemistry.

In summary, the results of this study highlight the potential of nanoporous CuO, CuO/Ag, and CuO/Ag/NiO-based composite materials as promising candidates for electrochemical applications, including glucose sensors. These materials hold great promise for the development of advanced electrochemical devices with enhanced performance, which could have significant implications in various fields, including biomedical engineering, energy storage, and environmental monitoring.

## Data Availability

The datasets used and/or analysed during the current study available from the corresponding author on reasonable request.
